# Dosimetric impact of gold markers implanted closely to lung tumors: a Monte Carlo simulation

**DOI:** 10.1120/jacmp.v15i3.4594

**Published:** 2014-05-08

**Authors:** Takehiro Shiinoki, Akira Sawada, Yoshitomo Ishihara, Yuki Miyabe, Yukinori Matsuo, Takashi Mizowaki, Masaki Kokubo, Masahiro Hiraoka

**Affiliations:** ^1^ Department of Radiation Oncology and Image‐applied Therapy Graduate School of Medicine, Kyoto University Kyoto; ^2^ Department of Therapeutic Radiology Graduate School of Medicine, Yamaguchi University Yamaguchi; ^3^ Department of Radiological Technology Faculty of Medical Science, Kyoto College of Medical Science Kyoto; ^4^ Department of Radiation Oncology Kobe City Medical Center General Hospital Hyogo; ^5^ Division of Radiation Oncology Institute of Biomedical Research and Innovation Hyogo Japan

**Keywords:** Monte Carlo simulation, gold marker, lung treatment planning, image‐guided radiotherapy (IGRT), dynamic tumor tracking, Vero4DRT

## Abstract

We are developing an innovative dynamic tumor tracking irradiation technique using gold markers implanted around a tumor as a surrogate signal, a real‐time marker detection system, and a gimbaled X‐ray head in the Vero4DRT. The gold markers implanted in a normal organ will produce uncertainty in the dose calculation during treatment planning because the photon mass attenuation coefficient of a gold marker is much larger than that of normal tissue. The purpose of this study was to simulate the dose variation near the gold markers in a lung irradiated by a photon beam using the Monte Carlo method. First, the single‐beam and the opposing‐beam geometries were simulated using both water and lung phantoms. Subsequently, the relative dose profiles were calculated using a stereotactic body radiotherapy (SBRT) treatment plan for a lung cancer patient having gold markers along the anteriorposterior (AP) and right‐left (RL) directions. For the single beam, the dose at the gold marker‐phantom interface laterally along the perpendicular to the beam axis increased by a factor of 1.35 in the water phantom and 1.58 in the lung phantom, respectively. Furthermore, the entrance dose at the interface along the beam axis increased by a factor of 1.63 in the water phantom and 1.91 in the lung phantom, while the exit dose increased by a factor of 1.00 in the water phantom and 1.12 in the lung phantom, respectively. On the other hand, both dose escalations and dose de‐escalations were canceled by each beam for opposing portal beams with the same beam weight. For SBRT patient data, the dose at the gold marker edge located in the tumor increased by a factor of 1.30 in both AP and RL directions. In clinical cases, dose escalations were observed at the small area where the distance between a gold marker and the lung tumor was ≤ 5 mm, and it would be clinically negligible in multibeam treatments, although further investigation may be required.

PACS number: 87.10.Rt

## I. Introduction

In radiation therapy (RT), tumor motion during respiration results in signifi cant geometric and dosimetric uncertainties in the dose delivery to the thorax and abdomen. Conventionally, large internal margins (IMs) are needed to fully cover the geometric changes that occur during free breathing; these large IMs may result in toxicity to healthy tissue. As techniques to manage respiratory‐induced tumor movement, breath‐hold,^1^, ^2^ respiratory gated RT,^3^, ^4^, ^5^ and fourdimensional techniques^(6)^ are effective in reducing the IM, resulting in a lower dose to the normal tissue and, thus, a lower risk of complications.

**Figure 1:**
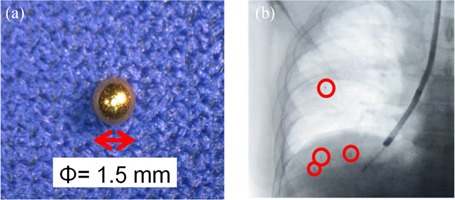
Photographs of (a) a gold marker with a diameter of 1.5 mm (courtesy of Olympus Medical Systems Corporation. Japan) and (b) an X‐ray fluoroscopy image of four gold markers implanted in a lung. The photon mass attenuation coefficient of gold markers is much larger than that of normal tissue.

A four‐dimensional, image‐guided radiotherapy system, Vero4DRT, was recently developed by Mitsubishi Heavy Industries, Ltd. (Tokyo, Japan) and BrainLAB (Feldkirchen, Germany), in collaboration with Kyoto University and the Institute of Biomedical Research and Innovation.^(7)^ The system has a gimbaled X‐ray head composed of a compact 6 MV linac with a C‐band, klystron‐based accelerator.^(8)^ We are developing an innovative dynamic tumor tracking irradiation technique using gold markers implanted around the tumor as a surrogate signal (Fig. 1), a real‐time marker detection system, and the gimbaled X‐ray head.

Several investigators have evaluated the dosimetric impact of gold seeds and various fiducial markers in the water phantom for photon or proton beams in image‐guided radiotherapy (IGRT).^9^, ^10^, ^11^ Our group has aimed to archive dynamic tumor tracking irradiation using several gold markers for lung cancers.^12^, ^13^ Therefore, it is important to understand the dose variation near the gold markers in the lung, and few studies have been reported.

The purpose of this study was to simulate the dose variation near a gold marker in a lung irradiated by a photon beam using the Monte Carlo method. First, the single‐beam and opposing‐beam geometries of the Vero4DRT system were simulated using both water and lung phantoms, respectively. Then, the dose variations near the gold marker were computed. Subsequently, relative dose profiles along the anterior‐posterior (AP) and right‐left (RL) directions of the computed tomography (CT) were calculated using a stereotactic body radiotherapy (SBRT) treatment plan for a lung cancer patient having gold markers.

## II. MATERIALS AND METHODS

### A. Monte Carlo simulation

A 6 MV photon beam delivered from the Vero4DRT system was simulated using the BEAMnrc accelerator, and DOSXYZnrc codes.^14^, ^15^ The linear accelerator head in the Vero4DRT system was simulated using the BEAMnrc code. The modeled linear accelerator head is composed of a compact C‐band 6 MV accelerator tube, a target, a primary collimator, a flattening filter, a monitor chamber, fixed secondary collimators, and a multileaf collimator. The description of the linear accelerator, such as the geometries and the materials of each component, were provided by the manufacturer.^(16)^

The field size was set to $5.0\times 5.0\;{\text{cm}}^{2}$. The simulation time was 40 hours on a PC having an Intel Xeon Quad Core 2.4 GHz with 16 GB memory. For the transport parameter of EGSnrc, the electron cutoff energy, ECUT, was set to 0.521 MeV, while the photon cutoff energy, PCUT, was set to 0.01 MeV. The generated phase‐space file had $5\times 10^{8}$ particles and the particles were recycled up to 25 times.^(17)^ All of simulation was performed without variance reduction techniques. The generated phase‐space file was used to calculate the percent depth dose and the off‐center ratio with a voxel size of $5.0\times 5.0\times 5.0\;{\text{mm}}^{3}$ using a water phantom. $5.0\times 10^{8}$ photon histories delivered to the water phantom were employed to reduce the dose statistical uncertainty ≤1.5% in the irradiation field.

On the other hand, the corresponding dose measurement was performed using our Vero4DRT system. Then, the differences between the simulated and measured doses were calculated along the beam axis and its vertical (lateral) axis, respectively.

### B. Simple geometric model of one gold marker and photon beam

**Figure 2:**
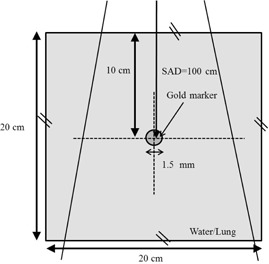
A geometric scheme shows a gold marker of 1.5 mm in diameter with single photon beam geometry for water phantoms. The gold marker was positioned at the isocenter which was located at depth of 10 cm from the surface in a water phantom and a lung phantom, respectively. The broken crosshair lines represent the relative dose profiles computed by the Monte Carlo simulation.

Figure 2 shows a simple geometric model having a gold spherical marker of 1.5 mm in diameter (FMR‐201CR; Olympus Co., Ltd., Tokyo, Japan) inside a water phantom $\left(20\times 20\times 20\;{\text{cm}}^{3}\right)$ with a single photon beam. The gold marker was positioned at the isocenter, which was located at a depth of 10 cm from the water surface.

Irradiation by a single photon beam was simulated with a source‐to‐axis distance (SAD) of 100 cm and a field size of $5.0\times 5.0\;{\text{cm}}^{2}$. The voxels outside the gold markers had a resolution of $0.20\times 0.20\times 0.20\;{\text{mm}}^{3}$, and those inside the gold marker had a resolution of $0.15\times 0.15\times 0.15\;{\text{mm}}^{3}$. The relative dose profiles along the beam axis and its perpendicular axis passing through the center of the gold marker were calculated (broken lines in Fig. 2).

The opposing portal beam along the beam axis was aligned with the gold marker in the field of $5\times 5\;{\text{cm}}^{2}$. The ratio of the beam weights was set to 1:3. The relative dose profile at a depth of 10 cm and the relative dose profile along the beam axis were calculated. Each dose was normalized to the simulated dose at the isocenter with no gold markers in the water phantom.

Subsequently, a similar simulation using a lung phantom was performed in the same manner.

For the simulation of simple geometry, the total number of photon histories was ranged from $5\times 10^{8}$ to $6\times 10^{8}$ to reduce the dose statistical uncertainty ≤1.5% in the region of interest. The total simulation run times were 66‐112 hours.

### C. Patient's CT based geometry model with SBRT

An SBRT patient having gold markers closely implanted to the lung tumor was enrolled. For the treatment planning, the whole lung was scanned under an end‐exhalation, breath‐hold condition with 2.5 mm thickness using a 16 slice CT scanner (LightSpeed RT; GE Healthcare, Waukesha, WI).

The treatment plan was created using iPlan RT dose 4.5.1 treatment planning system (BrainLAB). Seven small fields were created at gantry angles of 15°, 175°, 220°, 270°, 295°, 315°, and 335°. Four fields of them were set to noncoplanar beam arrangement, and the others were set to coplanar beam arrangement. The prescribed dose was 4800 cGy in four fractions. This plan was designed for the Vero4DRT system. The spatial resolution of the multileaf collimator was 5.0 mm at the isocenter.

The CTCREATE program taken from the DOSXYZnrc was used to convert the lung patient CT data at end‐exhalation to materials and mass densities with a $2.0\times 2.0\times 2.5\;{\text{mm}}^{3}$ of simple geometric model.^18^, ^19^ The streaking artifacts in the CT data were partially mitigated by assigning International Commission on Radiation Units and Measurements (ICRU) lung and tissue to the voxels.^(20)^

The above clinical plan was simulated in the DOSXYZnrc code using a phase space file commissioned for the Vero4DRT. The number of photon histories was $9.0\times 10^{8}$, while the sizes of phase space files were 2.6‐3.3 GB for each field. The total simulation run times were 315 hours. A Monte Carlo simulation was iteratively performed until the total statistical error was less than 1.5% in the region of interest.

## III. RESULTS & DISCUSSION

In this study, Monte Carlo simulation was performed to estimate the radiation dose around a gold marker irradiated by a photon beam. The one significant advantage of the simulation is that it allows dose calculation inside the gold marker, as well as at the edge between the gold marker and the phantom, although the measurement by a chamber is impossible.

The geometric arrangement of the beam and the gold markers in the Monte Carlo simulation helps to avoid human errors in positioning the gold marker and the chamber in the measurement setup.

In patient's CT‐based geometry model with SBRT, Monte Carlo simulation was performed using end‐exhalation CT. Fujisaki et al.^(21)^ has reported that the average lung density at shallow exhalation and free breathing were equivalent to 0.23, and 0.22g/cc, respectively; therefore, the difference between dose calculated using the end‐exhalation phased CT and free‐breathing CT was very small.

**Figure 3:**
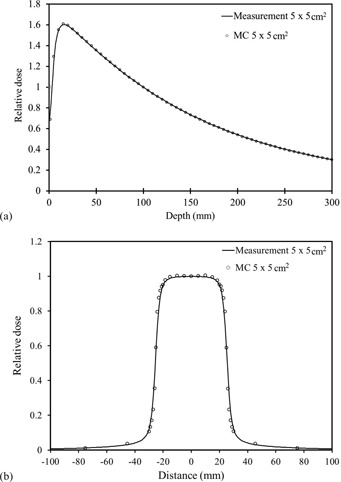
The simulated and the measured profiles of (a) percent depth dose and (b) off‐center ratio at a depth of 10 cm with a field size of $5.0\times 5.0\;{\text{cm}}^{2}$ with no gold marker. The simulated dose along the beam axis beyond the buildup point agreed with measured dose to within 1.0% in (a) and the simulated lateral dose agreed within 1.3% except around the penumbra in (b).

Figure 3 shows that the simulated and measured percent depth dose and off‐center ratio at a depth of 10 cm for a field size of $5.0\times 5.0\;{\text{cm}}^{2}$ with no gold marker. The simulated dose along the beam axis beyond the buildup point agreed with the measured dose within an error of 1.0%, and the simulated lateral dose agreed within 1.3%, except around the penumbra.

**Figure 4:**
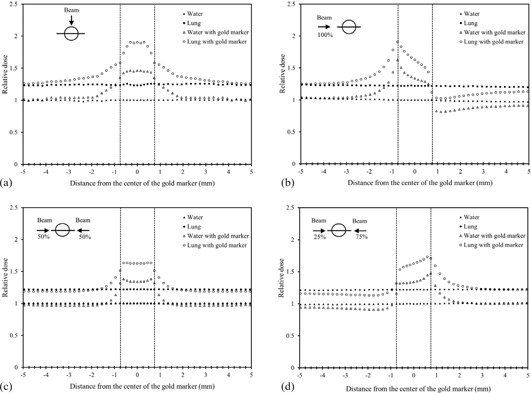
The relative dose profiles with and without the gold marker for the single‐beam and the opposing portal beam in a water and lung phantom. (a) The dose at the gold marker‐phantom interface laterally along the perpendicular to the beam axis increased by a factor of 1.35 in the water phantom and 1.58 in the lung phantom, respectively. (b) The entrance dose at the gold marker‐phantom interface along the beam axis increased by a factor of 1.63 in the water phantom and 1.91 in the lung phantom, while the exit dose at the gold marker‐phantom increased by a factor of 1.00 in the water phantom and 1.12 in the lung phantom, respectively. (c) For the opposing portal beams with the same beam weight, the dose at the gold marker‐phantom interface along the beam axis increased by a factor of 1.31 in the water phantom and 1.51 in the lung phantom, respectively. (d) For the opposing portal beams with a beam weight ratio of 1:3, the entrance dose at the gold marker‐phantom interface along the beam (weight=1) axis increased by a factor of 1.16 in the water phantom and 1.31 in the lung phantom, while the entrance dose at the gold marker‐phantom interface along the beam (weight=3) axis increased by a factor of 1.41 in the water phantom and 1.71 in the lung phantom, respectively.

Figures 4(a) to (d) show the relative dose profiles with and without a gold marker for the single and opposing portal beams in the water and lung phantoms. For the single beam, the dose at the gold marker‐phantom interface laterally along the perpendicular to the beam axis increased by a factor of 1.35 in the water phantom and 1.58 in the lung phantom, respectively (Fig. 4(a)). The entrance dose at the gold marker‐phantom interface along the beam axis increased by a factor of 1.63 in the water phantom and 1.91 in the lung phantom, while the exit dose at the gold marker‐phantom increased by a factor of 1.00 in the water phantom and 1.12 in the lung phantom, respectively (Fig. 4(b)). The above dose escalation was observed within about 5 mm off the edge from the phantom to the marker. On the other hand, the dose de‐escalation occurred within about 5 mm off the edge from the marker to the phantom. These were mainly due to the photoelectric effect near the interface of the gold marker.

For the opposing portal beams with the same beam weight, the dose at the gold markerphantom interface along the beam axis increased by a factor of 1.31 in the water phantom and 1.51 in the lung phantom, respectively (Fig. 4(c)). When the gold marker was irradiated by two opposing beams, the dose escalation and dose de‐escalation were canceled by the opposing beams. As a result, the dose escalations became smaller than those for the single beam. Figure 4(d) shows the dose profiles for the opposing portal beams with a beam weight ratio of 1:3. The entrance dose at the gold marker‐phantom interface along the beam (weight=1) axis increased by a factor of 1.16 in the water phantom and 1.31 in the lung phantom, while the entrance dose at the gold marker‐phantom interface along the beam (weight=3) axis increased by a factor of 1.41 in the water phantom and 1.71 in the lung phantom, respectively (Fig. 4(d)). The dose escalation occurred within about 3 mm from the gold marker to the phantom.

Chow and Grigorov^(9)^ have represented the dose escalation and dose de‐escalation information around a gold seed in the water phantom by performing a Monte Carlo simulation for Varian 21EX linear accelerator. The relative dose ranged from 0.88 to 1.64 at the edge between the gold seed and the water. Our study has demonstrated the similar results. Furthermore, dose escalation and dose de‐escalation information in the lung phantom was observed. The dose variations in the lung phantom were larger than those in the water phantom (Figs. 4(a) to (d)). These variations will be derived by the backscatter of secondary electrons from the gold marker and the lower mass density of lung.

**Figure 5:**
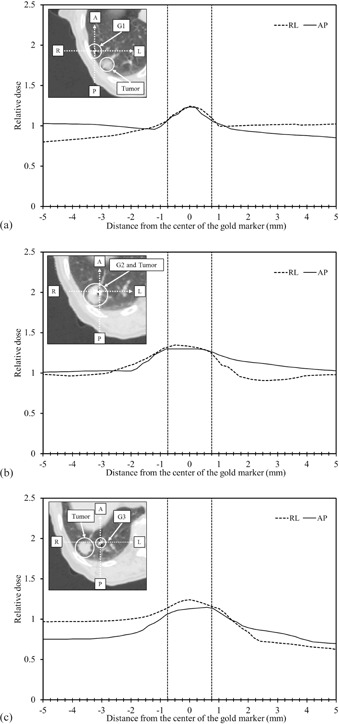
Three axial images in the superior‐inferior direction. One of three implanted gold markers (G1, G2, and G3) can be seen in each image, and the corresponding AP and RL lines are shown as broken lines. Hot spots were observed at G2, while they were not observed at G1 and G3. The dose escalation near the gold marker surface was ≤5mm in both the AP and RL directions.

Figure 5 shows three axial images in the superior‐inferior direction. Three gold markers along the superior‐inferior direction were labeled as G1, G2, and G3. For each gold marker, the relative doses along AP and RL directions were calculated. Each dose was normalized to the prescribed dose at the isocenter. The implanted gold markers (G1, G2, and G3) can be observed in each image, and the AP and RL lines via each gold marker are shown as broken lines. The distance between G1 (as well as G3) and the lung tumor was about 15 mm. G1 and G3 were located outside the planning target volume (PTV); G2 in the tumor. As discussed previously, dose escalations were observed when the distance between the gold marker and the tumor was within 5 mm. Therefore, dose escalations outside the gold marker were rarely observed for G1 and G3 (Figs. 5(a) and (c)), whereas they were observed near G2 (Fig. 5(b)). For G2, the dose at the gold marker edge increased by a factor of 1.30 in the RL and AP directions. However, the dose escalation near the gold marker surface was less than 5 mm and the volume was less than 65.4 mm^3^ in the lung. According to the ICRU report 50,^(22)^ a hot spot is defined to be a volume outside the PTV that receives a dose larger than 100% of the specified PTV dose. The hot spot is considered clinically meaningful only if the diameter of the volume exceeded 15 mm. The dose escalations near gold markers G1 and G3 were rarely observed; and therefore, they will be clinically negligible for the lung.

Recently, there has been strong interest in treating mobile tumors in the pelvis, abdomen, and thorax. The use of fiducial markers to manage organ motion has been widely reported, with no consideration for the dose escalation and dose de‐escalations that fiducial markers can cause.^23^, ^24^, ^25^ Our results provided dosimetric data such as relative doses and positions of dose escalation and dose de‐escalations around a gold marker.

## IV. CONCLUSIONS

Our simulation has demonstrated the dosimetric impact near a gold marker in lung irradiated by a 6 MV photon beam. The simulation results provided with dosimetric data, including relative doses and positions of dose escalation and dose de‐escalation near a gold marker under different beam geometries, as well as a clinical geometry based on CT images of a patient. In clinical cases, dose escalations were observed at the small area where the distance between a gold marker and the lung tumor was ≤5mm, and it would be clinically negligible in multibeam treatments, although further investigation may be required.

## ACKNOWLEDGMENTS

This research was supported by the Japan Society for the Promotion of Science (JSPS) through its Funding Program for World‐Leading Innovative R&D on Science and Technology (FIRST Program).
